# Diagnosis, prognosis and bioinformatics analysis of lncRNAs in hepatocellular carcinoma

**DOI:** 10.18632/oncotarget.21329

**Published:** 2017-09-28

**Authors:** Meiyu Dai, Siyuan Chen, Xiaomou Wei, Xuan Zhu, Fang Lan, Shengming Dai, Xue Qin

**Affiliations:** ^1^ Department of Clinical Laboratory, First Affiliated Hospital of Guangxi Medical University, Nanning, Guangxi 530021, China; ^2^ Department of Clinical Laboratory, Fourth Affiliated Hospital of Guangxi Medical University, Liuzhou, Guangxi 545005, China

**Keywords:** hepatocellular carcinoma, lncRNA, diagnosis, prognosis, expression

## Abstract

This study aims to comprehensively analyze the diagnosis and prognosis of lncRNAs in hepatocellular carcinoma (HCC). From the Gene Expression Omnibus database, we screened out and analyzed the differently expressed lncRNAs and related mRNAs using bioinformatics methods. The expressions of lncRNAs were validated in tumor tissues, cell lines and the Cancer Genome Atlas database. At the same time, we also conducted an exploratory analysis on the diagnostic and prognostic ability of lncRNAs in HCC. In this study, we found that most of the targeted mRNAs promote the biological process of cell division, cellular component of nucleoplasm, molecular function of protein binding, which were significantly associated with 12 KEGG pathways. LncRNA CRNDE and LINC01419 also had significant diagnostic value in HCC. In particular, the sensitivity, specificity and area under the curve of CRNDE were 71.0%, 87.1% and 0.701 (95% CI: 0.543-0.860), respectively. In addition, the high expression of CRNDE and GBAP1 predicated poor prognosis, while the high expression of LINC01093 suggested the opposite outcome. Through the comprehensive analysis of lncRNAs, it provided an important reference for the early diagnosis, prognosis evaluation, pathogenesis and targeted therapy of HCC.

## INTRODUCTION

Primary liver cancer, also called hepatocellular carcinoma (HCC), is one of the most common and aggressive malignant tumors all over the world. HCC is the third biggest cause of cancer related deaths worldwide [1]. In China, there are about 360,000 new cases and 350,000 deaths each year [2]. The HCC patients are 40 to 50 years old on average. Male has significantly higher incidence of HCC over female [3]. However, etiology and pathogenesis of HCC have not been fully determined. At present, it is thought to be related to liver cirrhosis, viral hepatitis, environmental factors, and chemical carcinogens such as aflatoxin. These diseases are mostly diagnosed at the late stage for the lack of specific clinical manifestations and over 80% of these patients are often left poor prognosis. Many studies reported that the five-year survival rate of HCC was less than 5%. Although some articles reported some serum biomarkers for early diagnosis, these results were not very satisfactory [4–6]. Therefore, it is urgent to find new biomarkers for the early diagnosis, treatment and prognosis evaluation of HCC.

Non-coding RNA is one of the hottest areas of life science research in the past ten years. Non-coding RNA, as its name implies, is the RNA that does not encode proteins. It mainly includes rRNA, tRNA, snRNA, snoRNA, siRNA, piRNA and microRNA etc. In addition, it also includes long non-coding RNA (lncRNA), which is longer than 200 nucleotides [7]. At present, there are many important researches about lncRNAs in the diagnosis and prognosis of various cancers, such as glioblastoma multiforme [8], lung cancer [9, 10], ovarian cancer [11, 12], multiple myeloma [13] and lymphoma [14]. Therefore, lncRNAs, as an important guiding role for clinicians, have crucial diagnostic and prognostic value in clinical practice. However, their species, quantity, function and mechanism are still completely unclear. Most of the lncRNAs in the process of differentiation and development of the tissue have obvious temporal and spatial specificity. They have a distinct expression in cancer and other diseases. Therefore, these lncRNAs are also thought to be associated with the cancerization or tumor suppression, and the level of expression can contribute to the evaluation of the diagnosis and prognosis for cancers [15, 16].

With the improvement of chip technology, high-throughput sequencing and other gene detection means, as well as the advances in bioinformatics, computational models and other analysis methods, more and more differentially expressed genes are found to be associated with various cancers. Some aberrant lncRNAs are found to be associated with tumor cell growth, invasion, proliferation, metastasis, differentiation and apoptosis [17–20]. In our study, we screened out the differentially expressed lncRNA profiles between HCC tissues and adjacent normal tissues by the integrated mining of the Gene Expression Omnibus (GEO). Then we carried out a comprehensive bioinformatics analysis, experimental verification and survival analysis using the Cancer Genome Atlas (TCGA) database. In the meantime, we predicted their regulatory mechanism and pathway in HCC and analyzed their diagnostic and differential diagnostic performance to provide an important reference for the early diagnosis and targeted therapy of HCC.

## RESULTS

### Differentially expressed lncRNA and mRNA profiles

In this comprehensive analysis, a total of six datasets were incorporated (GSE57555, GSE19665, GSE29721, GSE33006, GSE46408 and GSE57957). With reference to p ≤ 0.05 and fold change ≥2.0, we screened out differentially expressed lncRNAs and mRNAs in the six datasets respectively. Then Robust Rank Aggregation (RRA) method was used to integrate all the differentially expressed lncRNAs of the six datasets and synthetically calculate the significantly differentially expressed lncRNAs and mRNAs. In the end, 14 lncRNAs (including 5 up-regulated lncRNAs: CRNDE, LINC01419, PTTG3P, GBAP1, CHL1-AS1 and 9 down-regulated lncRNAs: A2M-AS1, CYP2B7P, H19, HAND2-AS1, LINC01093, LINC01146, LOC101928505, LOC200772, NAPSB) and 607 mRNAs (including 521 mRNAs related to up-regulated lncRNAs, 105 mRNAs related to down-regulated lncRNAs and 19 mRNAs related to both up-regulated and down-regulated lncRNAs) were selected as the most significantly differential and relevant genes.

### qRT-PCR validation of the lncRNA profiles

#### Validation in tumor tissue samples

**Figure 1:**
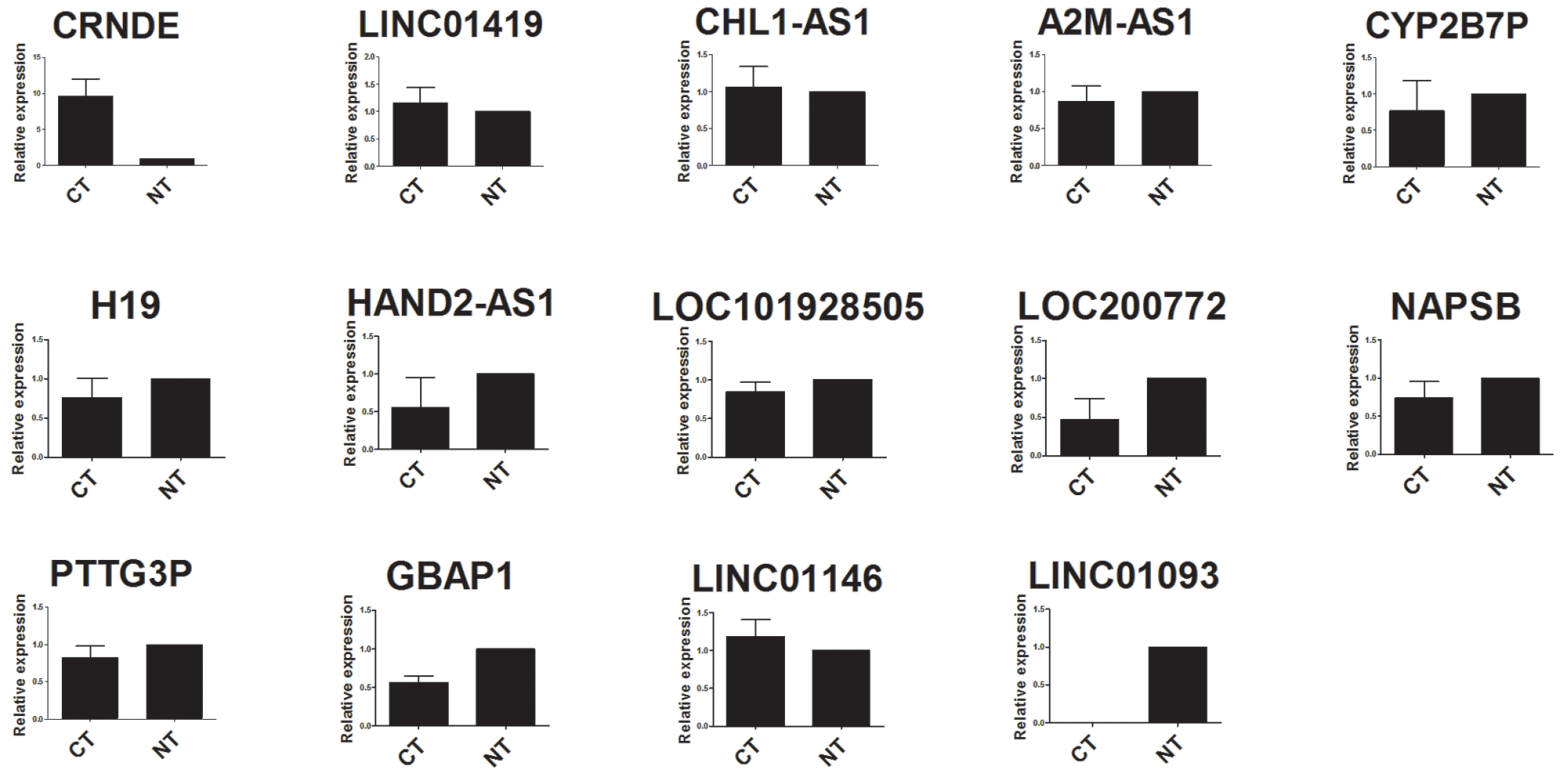
qRT-PCR analysis of fourteen lncRNAs expression in HCC tissues and adjacent normal tissues CT: HCC tissues; NT, adjacent normal tissues.

Validation between HCC tissues and adjacent normal tissues was performed in all the fourteen lncRNAs by quantitative real-time PCR (qRT-PCR). Although not all the results existed significant difference, ten out of fourteen lncRNAs were basically consistent with the microarray data in the relative expression tendency. Three lncRNAs, PTTG3P, GBAP1 and LINC01146, were inconsistent with the microarray data, which may attribute to the difference of testing methods, the limitation of computing method or the limited number of samples mentioned in many similar studies [21–23]. In addition, lncRNA LINC01093 could be detected only in adjacent normal tissues, and the expression level of HCC tissues was too low to detect. This expression tendency was also consistent with the microarray data. The details were shown in Figure 1.

#### Validation in HCC cell lines

In order to make the validation results of lncRNAs more comprehensive and credible, we carried out the validation in tumor cell lines in addition to the experimental validation of the tumor tissue samples by qRT-PCR. The results of validation and the microarray data were basically the same, except for three lncRNAs (CHL1-AS1, HAND2-AS1 and LINC01093) that could not be detected. The detailed expression level was shown in Supplementary Figure 1. Additionally, we performed the same assay for cell culture supernatants. There were five lncRNAs (CRNDE, LINC01419, A2M-AS1, LINC01146 and LOC101928505) that were detected in the supernatant of cell culture, and the results were basically consistent with the results of cell detection. The detailed expression level was shown in Supplementary Figure 2.

### LncRNA-mRNA co-expression network

**Figure 2:**
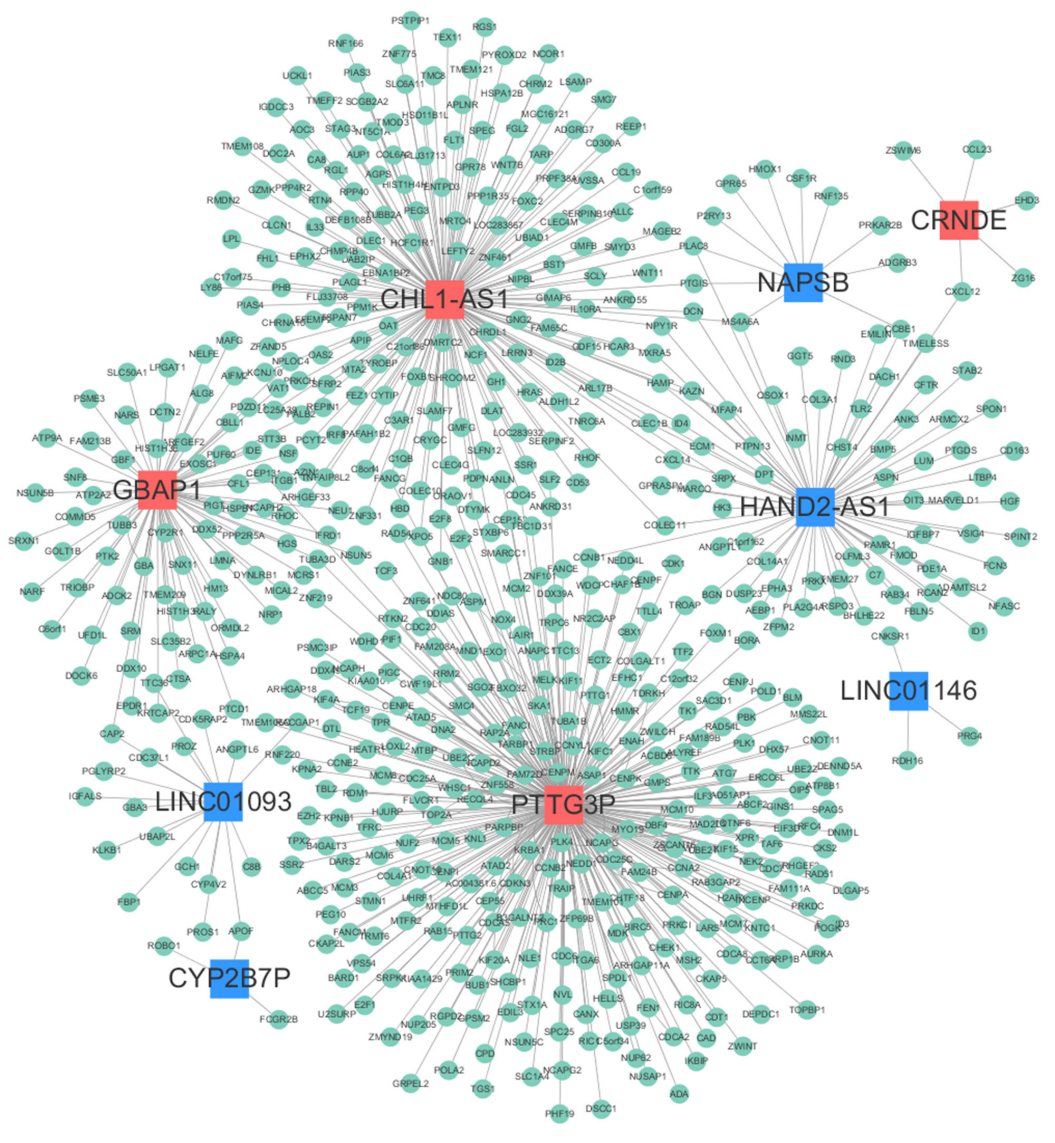
LncRNA-mRNA co-expression network Red nodes represent up-regulated lncRNAs, blue nodes represent down-regulated lncRNAs, and green nodes represent mRNAs.

In order to explore which lncRNA and mRNA played an important regulatory role in the HCC biological process, we constructed the lncRNA-mRNA co-expression network on the basis of the correlation coefficient between lncRNAs and mRNAs screened from microarray data. Finally, we found nine of the fourteen lncRNAs had obvious correlation with the 607 mRNAs. Among them, lncRNA CHL1-AS1, GBAP1, PTTG3P and HAND2-AS1 were the main control centers in the network diagram. The details of regulatory information were shown in Figure 2.

### GO and KEGG pathway analyses

**Figure 3:**
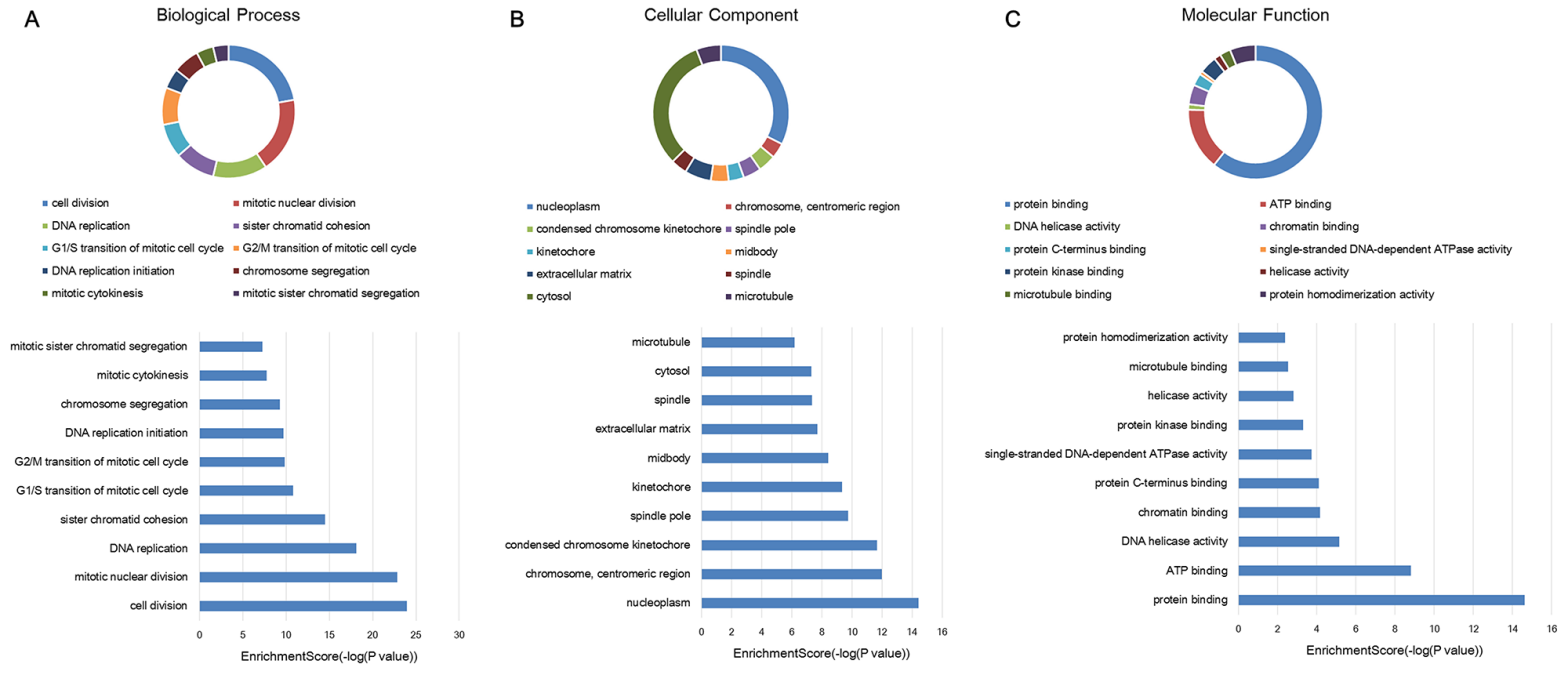
GO enrichment analysis of target genes associated with lncRNA profiles Proportional distribution and enrichment in **(A)** biological process, **(B)** cellular component, **(C)** molecular function.

**Figure 4:**
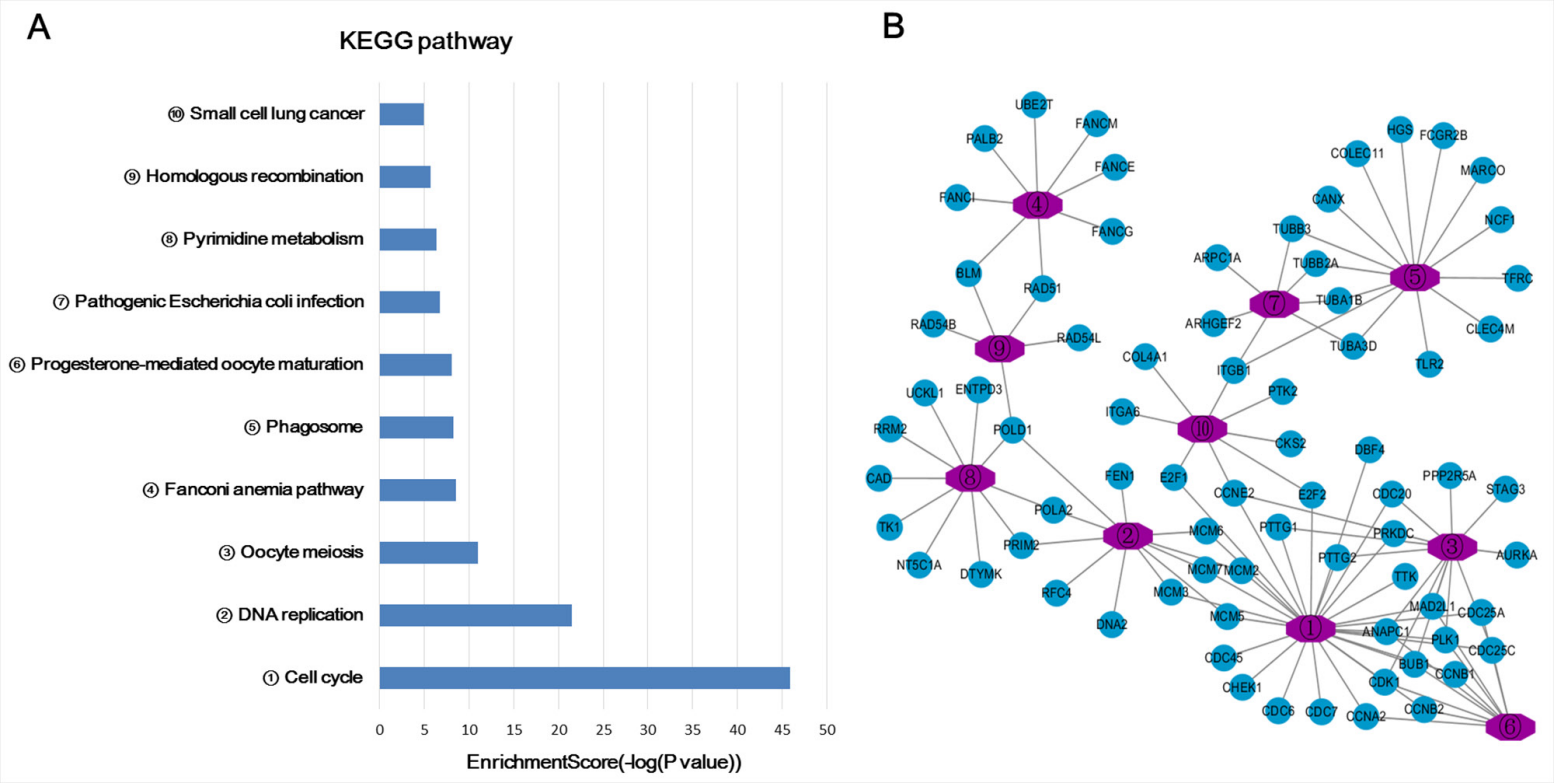
KEGG pathway enrichment analysis of target genes associated with lncRNA profiles **(A)** Bar chart of the KEGG pathway enrichment analysis. **(B)** A network of genes related to the pathway.

In this study, the gene ontology (GO) and KEGG pathway analyses were performed in all the mRNAs related lncRNA profiles. Since most of mRNAs were positively related to the lncRNAs (534 mRNAs in positive correlation and 73 mRNAs in negative correlation), we do not carry out separate enrichment analysis. GO analysis could classify genes into three categories, including biological process (BP), cellular component (CC) and molecular function (MF). From the top ten of the count number proportion and category enrichment analysis, cell division and mitotic nuclear division in the BP, nucleoplasm and cytosol in the CC, protein binding and ATP binding in the MF accounted for most of the proportion and the most significant correlation with HCC (Figure 3). In the enrichment analysis of KEGG pathway, a total of 12 pathways were significantly associated with the targeted mRNAs, of which cell cycle signal pathway was the most obvious. The details were shown in Figure 4.

### Diagnostic value of lncRNAs in serum

**Figure 5:**
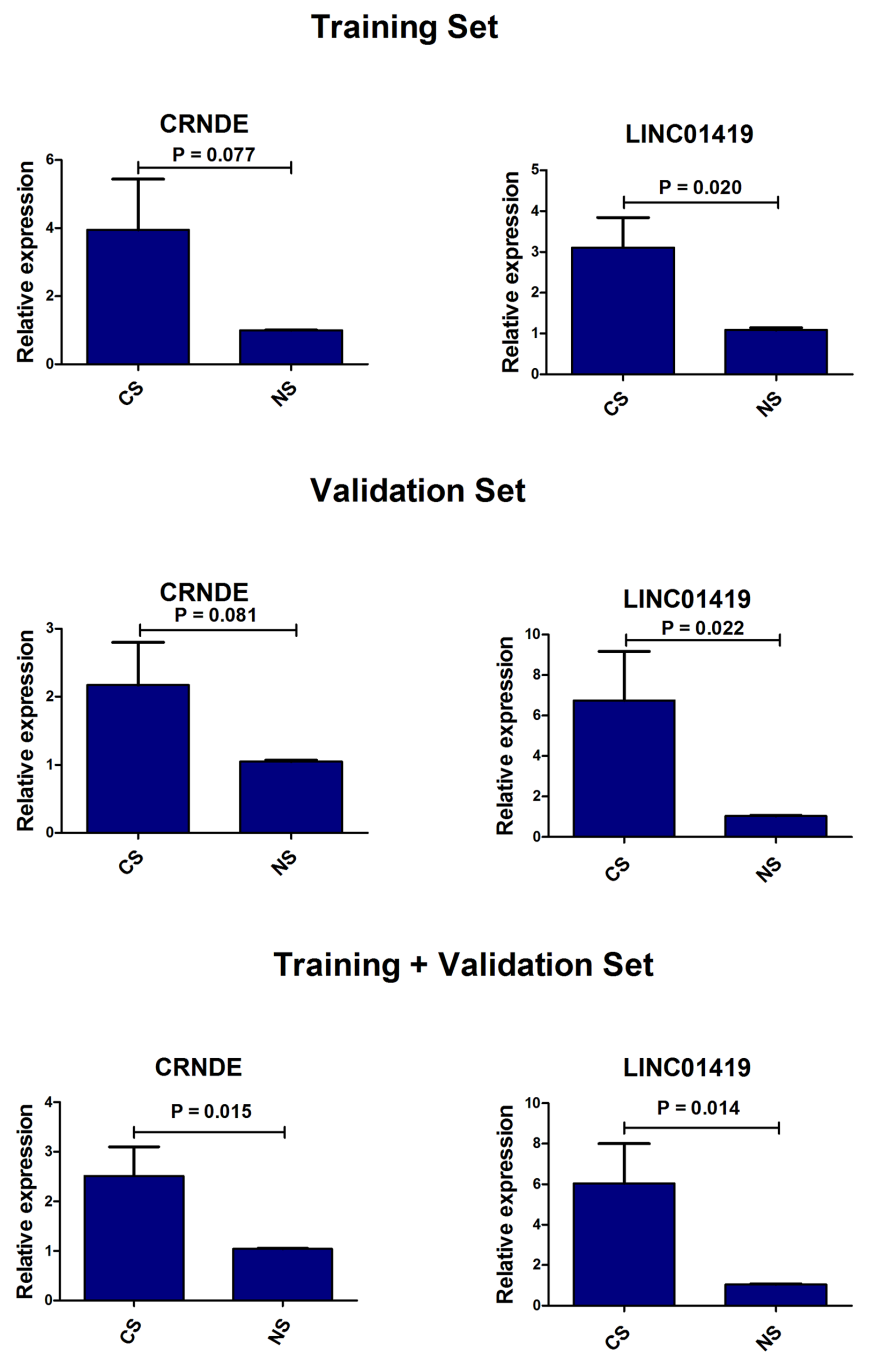
The expression levels of CRNDE and LINC01419 in serum samples between HCC patients and health controls CS: serum samples of HCC patients, NS: serum samples of health controls.

In the previous experiments, we detected lncRNA in the supernatant of the cell culture, so we speculated that lncRNA might be detected in the serum of patients. First of all, we detected all the lncRNAs in the training set by qRT-PCR, and found that only two lncRNAs, CRNDE and LINC01419, could be detected in serum samples. Then we extended the sample size for further detection analysis. The results indicated that the expression levels of both lncRNA CRNDE and LINC01419 in HCC patients group were obviously higher than that in healthy group (Figure 5). There were no significant difference between the non-cancer group and the healthy group (CRNDE: p = 0.254; LINC01419: p = 0.124), or between the non-cancer group and the HCC group (CRNDE: p = 0.150; LINC01419: p = 0.855). In addition, we also evaluated the diagnostic value of the two lncRNAs between HCC patients and health controls. There was a certain auxiliary diagnostic significance for lncRNA CRNDE in HCC with the sensitivity, specificity and area under the curve (AUC) of 71.0%, 87.1% and 0.701 (95% CI: 0.543-0.860). Detailed data were shown in Table 1.

**Table 1:** The diagnostic accuracy of CRNDE and LINC01419

LncRNA	Sensitivity	Specificity	AUC	95% CI
**CRNDE**	0.710	0.871	0.701	0.543-0.860
**LINC01419**	0.484	0.968	0.522	0.350-0.694
**Combination**	0.677	0.903	0.674	0.512-0.837

### Validation and survival analysis in TCGA

**Figure 6:**
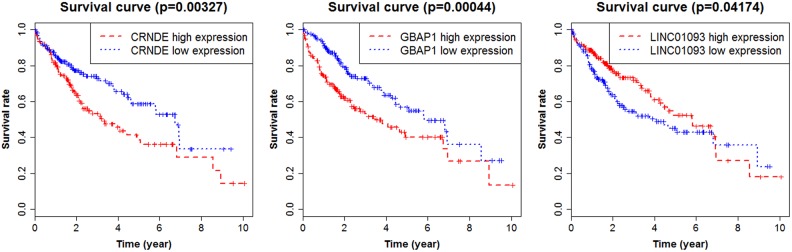
Survival curves showing the relationship between the lncRNAs and the overall survival rate

Transcript copy number of HCC was downloaded from the TCGA database to verify the expression levels of the lncRNA profiles. There were a total of 424 samples, of which 374 cases were HCC tissues and 50 cases were the adjacent normal tissues. By converting the gene symbols, we found lncRNA LOC101928505 was not in TCGA database, and another three lncRNAs (PTTG3P, LINC01419 and CHL1-AS1) were removed because the copy number was too low to be detected. Finally, ten lncRNAs could be verified. The results showed that the expression levels of nine lncRNAs were consistent with the microarray data from GEO database, except for one lncRNA A2M-AS1 with no statistical significance. In addition, we analyzed the survival rate of the nine lncRNAs. The results indicated that there was a significant correlation between three lncRNAs (CRNDE, GBAP1 and LINC01093) and the prognosis of HCC. The high expression of lncRNA CRNDE and GBAP1 suggested a poor prognosis, whereas the high expression of lncRNA LINC01093 suggested a good prognosis (Figure 6). In addition, we also performed a correlation analysis regarding patients’ age, gender and stage by Pearson's Chi-square Test (Table 2). The results indicated that patients’ age had a significant effect on the prognosis. They were not independent indicators in the clinical evaluation of the prognosis, and therefore they were used only as reference conditions. There was no significant correlation between the prognosis of HCC and other lncRNAs (Supplementary Figure 3).

**Table 2:** Correlation between lncRNA expression and HCC clinical features

Factor	CRNDE		*p* value	GBAP1		*p* value	LINC01093		*p* value
	High	low		High	low		High	Low	
**Age**			0.037^*^			0.013^*^			0.01^*^
**<61**	78	99		95	82		89	88	
**≥61**	106	87		128	65		129	64	
**Gender**			0.884			0.943			0.876
**Female**	60	61		73	48		71	50	
**Male**	127	125		153	99		150	102	
**Stage**			0.091			0.029^*^			0.580
**I-II**	122	138		147	113		152	108	
**III-IV**	51	38		62	27		55	34	

^*^p < 0.05: the values had statistically significant differences.

## DISCUSSION

With the rapid development of high-throughput sequencing technology and the wide application of related technologies, biomedical research has entered into the post-genomic era with an exponential increase in large-scale omics data. At this moment, biological computing and bioinformatics could help dig out the meaningful and regular genes from the complex data. In order to find out the important biological pathways that are related to biological processes, the researchers usually identify differentially expressed genes on the basis of a variety of statistics and calculation modes [24–27], and then perform the function enrichment analysis for differentially expressed genes so as to reveal and understand the basic molecular mechanism.

In our study, we finally screened 14 lncRNAs and 607 mRNAs, which were differentially expressed between HCC tissues and adjacent non-cancer tissues. Then GO and KEGG pathway enrichment analysis were performed in the differentially expressed targeted mRNAs. GO can be divided into three parts: molecular function, biological process and cellular component. Protein or gene can use the ID number or the sequence annotation method to find the corresponding GO number, and GO number can correspond to terms, which are the functional categories or cell location. In our study, the results of GO enrichment analysis indicated that most differentially expressed mRNAs were enriched in the biological process of cell division and mitotic nuclear division, cellular component of nucleoplasm and cytosol, molecular function of protein binding and ATP binding. There are specific genes that control cell division, differentiation, senescence and death within the cells [28, 29]. When there are abnormal cell differentiation, cell nucleus division or physiological imbalance, tumor and cancer will be formed. Cancer cells are a group of cells that have lost their normal physiological functions. It can be seen that these differentially expressed mRNA sets are closely related to the occurrence and development of cancers. KEGG pathway analysis of differentially expressed genes can be used to explain the results of the study. It is possible to find the KEGG pathway terms for the enrichment of differential genes and to find out which genes may be related to the changes of the cellular pathways. Our study indicated that 12 pathways were closely associated with the differently expressed mRNAs, of which cell cycle, DNA replication and Oocyte meiosis were the primary signal pathways. Different from the GO analysis, the results of KEGG pathway analysis are more indirect, because pathway is an interaction among proteins, and changes in pathway can be caused by the changes in the expression of proteins involved in the pathway or protein activity. The changes in the expression of mRNAs encoding these proteins were obtained through the microarray data. However, the expression from mRNA to protein is required to go through many processes, such as miRNA or lncRNA regulation, translation, modification, protein transport and so on. Therefore, we conducted lncRNA-mRNA co-expression network to further analyze the regulation relationship among them. Based on the above results, we could conclude that these lncRNA profiles played an important role in the development of HCC, which might be potential therapeutic targets for HCC patients.

In present study, we also performed validation experiment in tumor tissues and HCC cell lines by qRT-PCR. Although there was a little difference between the lncRNA expression and the results of the microarray data, most of the overall trends were consistent with the microarray data. The test results failed to match with the results of microarray due to the different detection methods or the calculation model error. It has been also reported that lncRNA was prone to rupture or instability because of its long length, so it may be mainly in the form of fragments in peripheral blood [30]. This also could result in distortion of the test results. At the same time, we detected the cell supernatant and found several detectable lncRNAs. There were also many reports indicating that lncRNAs were detectable in the blood [31, 32]. Therefore, we performed screening in the serum of HCC patients, non-cancer patients and healthy individuals to comprehensively and systematically explore the function and value of lncRNAs in HCC. First, we conducted a preliminary experiment and found that only lncRNA CRNDE and LINC01419 could be stably expressed in serum samples, and then we expanded the sample size to detect the two lncRNAs. Finally, we found that those two lncRNA were significantly higher expression levels in the HCC group than that in healthy group. At the same time, the diagnostic value of them was evaluated. It was found that CRNDE had high sensitivity and specificity in the diagnosis of HCC. Thus, it provides a new reference index for the diagnosis of HCC. Although the diagnostic sensitivity of lncRNA LINC01419 was not satisfactory, one study had shown that LINC01419 was highly expressed in early HCC tissues [33]. Therefore, LINC01419 was also valuable in the early diagnosis of HCC.

Diagnosis and prognosis are always two important indicators for various diseases [34, 35]. In this paper, we have made a study on the serological diagnostic index of HCC, and we also used the TCGA database to validate the differential expression levels of the fourteen lncRNAs. Then the survival analysis was performed for the lncRNAs that were consistent with the results of microarray data. We found that there was a significant correlation between the three lncRNAs (CRNDE, GBAP1 and LINC01093) and the survival rates of HCC patients. The high expression of lncRNA CRNDE suggested that the prognosis of HCC was poor. Most of the reports about CRNDE were associated with the diagnosis and prognosis of CRC and glioma, but few were reported related to HCC [36, 37]. The high expression of lncRNA GBAP1 and low expression of lncRNA LINC01093 also suggested poor prognosis of HCC patients. So far there has been very little study of the two lncRNAs. Therefore, further research on the two lncRNA functions and other research become particularly essential. In future research, we will continue to follow up its relevant research.

To sum up, our study selected 14 lncRNAs and 607 mRNAs by mining the GEO datasets. Then, a series of bioinformatics analysis were performed for the differentially expressed genes, such as GO analysis, KEGG pathway analysis, co-expression analysis, etc. In addition, the differential expressions of lncRNAs were validated in tumor tissues, HCC cell lines and TCGA database. The evaluation of serum's diagnostic value and survival analysis of HCC prognosis were also performed, which indicated that CRNDE and LINC01419 could be used as auxiliary diagnostic index of HCC and CRNDE, GBAP1 and LINC01093 could be used as prognostic indicators for HCC. The comprehensive analysis on the various functions of lncRNA could serve as an important reference for the early diagnosis, prognosis evaluation, pathogenesis and targeted therapy of HCC.

## MATERIALS AND METHODS

### HCC gene expression datasets

With the rapid development of high-throughput detection methods, a lot of public databases have been established, such as GEO (http://www.ncbi.nlm.nih.gov/geo/), TCGA (http://cancergenome.nih.gov/), Oncomine (https://www.oncomine.org/resource/main.html) and ArrayExpress (http://www.ebi.ac.uk/arrayexpress/) etc, among which GEO database is the largest and most comprehensive gene expression data resource. In our study, all the HCC related gene expression data were downloaded from the GEO database. There were four main criteria for data selection: (1) the specimens should be HCC tissues and matched para cancerous tissues; (2) each chip dataset must also contain lncRNAs and mRNAs; (3) the sample number of each dataset should not be less than three pairs; (4) If there were multiple articles sharing repeated data, only the largest sample size should be included. With the reference to the above screening criteria, six datasets were included in this paper for a comprehensive analysis (GSE57555, GSE19665, GSE29721, GSE33006, GSE46408 and GSE57957).

### Data processing and analyzing

Conventionally, six preprocessed series matrix files were downloaded from the GEO database. Some standardized procedures such as denoising, filtering, log2-transformation, were all performed in the six datasets. After data preprocessing, we finally found 20,209 mRNAs and 2,662 lncRNAs in GSE57555 dataset, 18,115 mRNAs and 2,009 lncRNAs in GSE19665 dataset, 18,115 mRNAs and 2,009 lncRNAs in GSE29721 dataset, 18,115 mRNAs and 2,009 lncRNAs in GSE33006 dataset, 18,229 mRNAs and 486 lncRNAs in GSE46408 dataset, 23,812 mRNAs and 1,740 lncRNAs in GSE57957 dataset. Then, with the standard of p ≤ 0.05 and fold change ≥ 2, all statistical analyses were performed in the six datasets to screen out lncRNAs and mRNAs with significantly differential expression. However, these datasets come from different platforms, and the results of these studies are often inconsistent. Therefore, it is necessary to integrate these data with appropriate statistical methods. In our study, we used the method of RRA which could integrate the results of independent data from different platforms. RRA not only achieves greater statistical power but also estimates the heterogeneity among the studies [38, 39]. The statistical operations were carried out using the Robust Rank Aggreg package of R with the standard of count number ≥ 2 and adjusted p value ≤ 0.05 (Version 3.2.3).

### Functional analyses for lncRNA profiles

The analysis of gene function enrichment refers to a large number of gene function sets that represent certain gene functional characteristics and biological processes. The common gene function database is composed of many gene function sets, such as GO, KEGG pathway, Reactome, Biocarta. In our study, GO and KEGG pathway analyses were used to elucidate the possible biological function and processes of the differentially expressed target genes by the online software of DAVID (https://david.ncifcrf.gov/). Since the FDR value of KEGG pathway terms was not significant, the p value ≤ 0.05 and count number ≥ 2 were used as the screening criteria for enrichment analysis. In addition, we constructed the lncRNA-mRNA co-expression network with the absolute value of Pearson's correlation coefficient no less than0.9. The co-expression network was drawn by Cytoscape (Version 3.3.0).

### Validation of the lncRNA profiles

#### Tissue samples of HCC patients

All samples used in this study were collected from the First Affiliated Hospital of Guangxi Medical University. All patients have signed informed consents and all experiments performed in our study were approved by the Institutional Review Boards at the First Affiliated Hospital of Guangxi Medical University. Meanwhile, all the experiments were carried out in accordance with the relevant standard operation instructions. A total of 15pairs of HCC tissues and adjacent normal tissues were used for the validation of the lncRNA profiles through qRT-PCR. The tissues were placed in liquid nitrogen immediately after surgery. The details of the HCC patients were shown in Supplementary Table 1.

#### Cell culture

Human normal liver cell (L-02) and HCC cells (LM3, SMMC-7721, MHCC97H and MHCC97L) were obtained from Shanghai Institutes for Biological Sciences. HCC cells (LM3, MHCC97H and MHCC97L) were cultured in the base medium of Dulbecco's modified Eagle's medium (DMEM) added with 10% fetal bovine serum (FBS, Gibco, Qualified, Australia Origin) and 100 units/ml antibiotics of penicillin-streptomycin (Invitrogen, Carlsbad, CA) in a humidified incubator with the atmosphere of 5% CO2 at temperature of 37°C. SMMC-7721 and L-02 were cultured in the base medium of Memorial Institute (RPMI) 1640 with the same condition as above. In addition, the supernatant was collected when the cells grew stably for 24 hours.

#### RNA extraction and qRT-PCR

The total RNA of all samples were extracted by the reagent of RNAiso Plus. A two-step method was adopted for quantification reverse transcription with 1 μg of total RNA. The SYBR Green method and 7500 Fast Real-Time PCR apparatus were both utilized in the qRT-PCR process. The PCR cycle process lasted for 10 min at 95 °C followed with 40 cycles of 15 s at 95°C, and then 1 min at 60 °C. The primers of lncRNA profiles and all reaction reagents were purchased from TAKARA BIO INC. The sequence of primers was shown in Supplementary Table 2. GAPDH gene was chosen as the reference gene. All the experiments were carried out in accordance with the corresponding instructions. The method of 2^-ΔΔCt^ was used to calculate the relative expression of lncRNAs.

#### TCGA database

TCGA is currently the largest database of cancer genetic information that contains unimaginable valuable information. At the same time, the TCGA database also contains detailed patient information. In our paper, the copy number data of HCC in TCGA database is used to verify the expression of lncRNAs identified from GEO database. In addition, we also performed a survival analysis of the validated lncRNAs to investigate their clinical significance.

### Serological diagnosis

A total of 90 serum samples were used for test, of which 30 specimens were obtained from HCC patients before surgery (part of them matched with tissues), 30 from the non-cancer patients including liver cirrhosis (6 cases), chronic HBV hepatitis (10 cases), hepatic cyst (8 cases) and hepatic fibrosis (6 cases), and 30 health controls with age and sex matched. Due to more lncRNAs to be tested, we randomly used six cases of HCC patients, non-cancer patients and healthy controls respectively as the preliminary experimental group to screen the differently expressed lncRNAs in serum and then expanded the sample size for further detection.

## SUPPLEMENTARY MATERIALS FIGURES AND TABLES



## Abbreviations

HCC: hepatocellular carcinoma
GEO: Gene Expression Omnibus
TCGA: the Cancer Genome Atlas
AUC: area under the curve
lncRNA: long non-coding RNA
RRA: Robust Rank Aggregation
qRT-PCR: quantitative real-time PCR
GO: gene ontology
BP: biological process
CC: cellular component
MF: molecular function
