# Supplementary material for: Diagnosis, prognosis and bioinformatics analysis of lncRNAs in hepatocellular carcinoma
Source: Oncotarget. 2017 Sep 28;8(56):95799–809. doi: 10.18632/oncotarget.21329 (PMC5707062; doi:10.18632/oncotarget.21329)
Supplement: Supplementary file 1 [file oncotarget-08-95799-s001.pdf]

Diagnosis, prognosis and bioinformatics analysis of lncRNAs in hepatocellular carcinoma

SUPPLEMENTARY MATERIALS

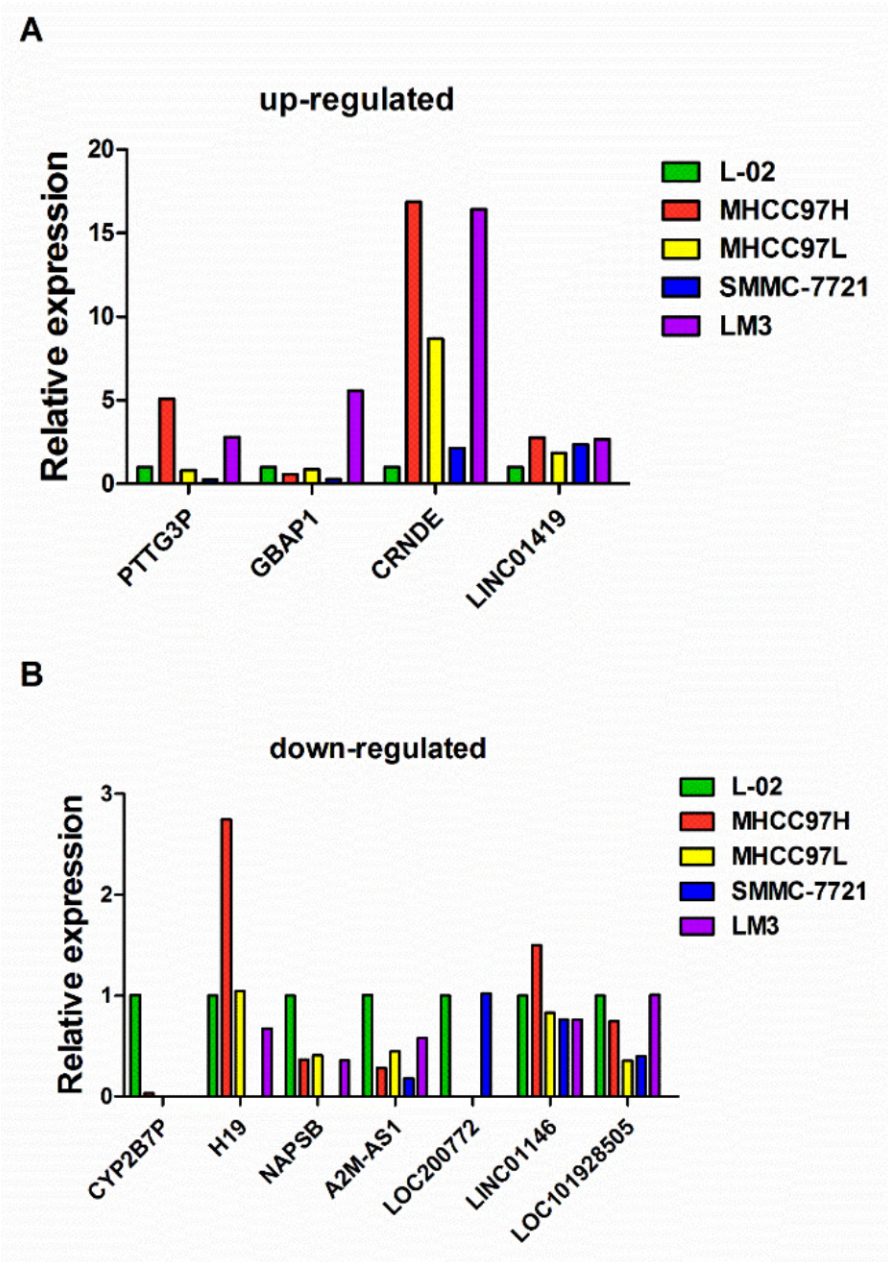

Supplementary Figure 1: The expression levels of LncRNAs in HCC cells and normal cell L-02. (A) The expression levels of up-regulated lncRNAs; **(B)** The expression levels of down-regulated lncRNAs.

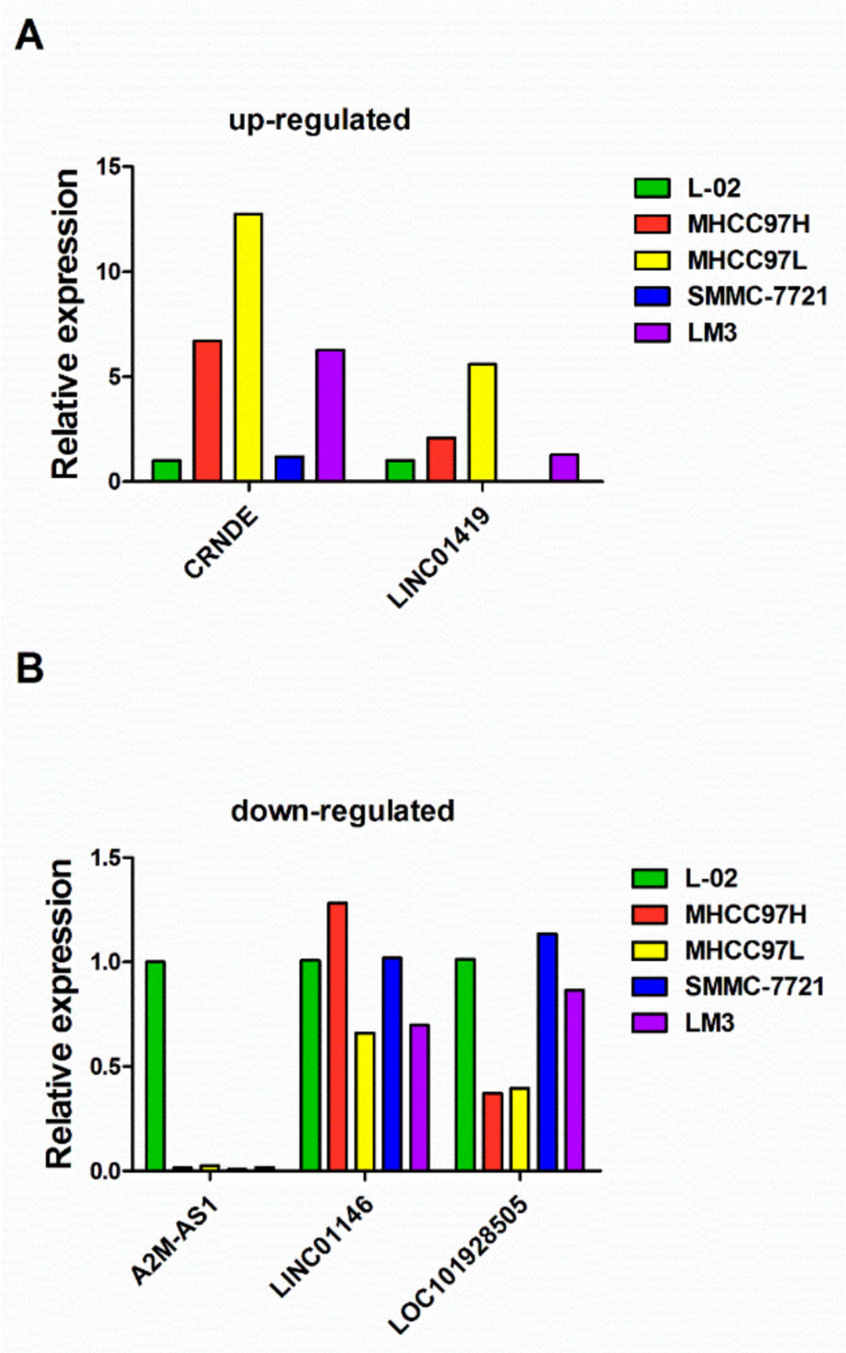

Supplementary Figure 2: The expression levels of LncRNAs in the supernatant of HCC cells and normal cell L-02. (A) The expression levels of up-regulated lncRNAs; **(B)** The expression levels of down-regulated lncRNAs.

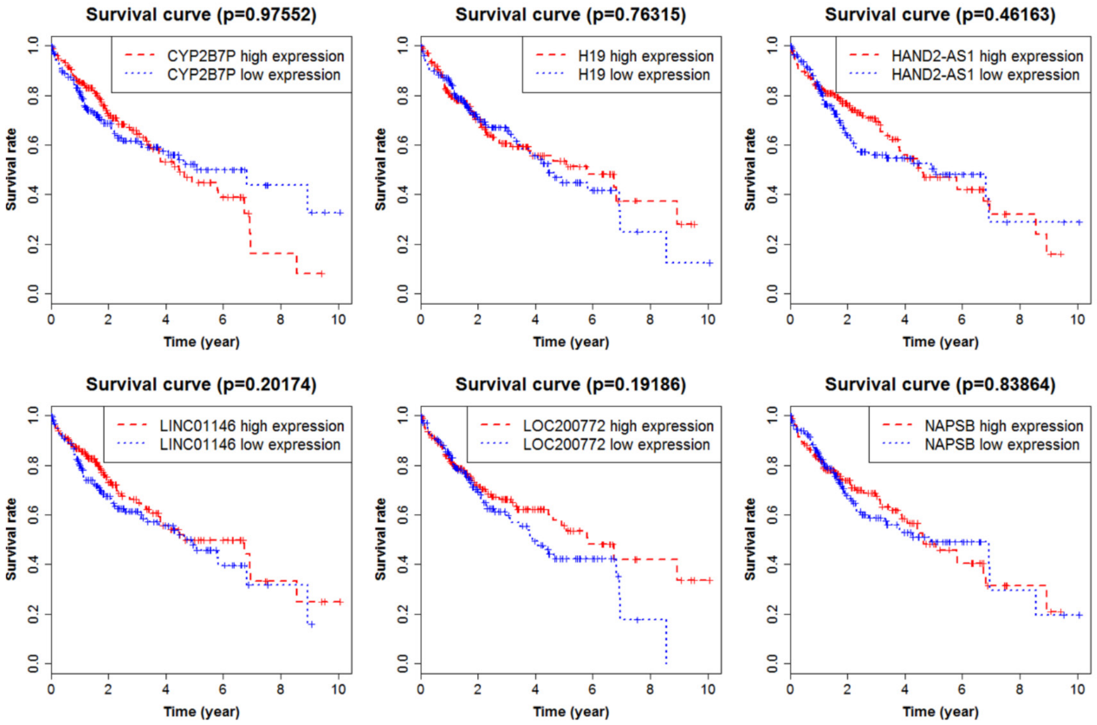

Supplementary Figure 3: Survival curves showing the relationship between the lncRNAs and the overall survival rate.

Supplementary Table 1: Clinical information of patients with tissue samples

Sample NO.	Gender	Age	HBV	Tumor morphology	Lymph node	Metastasis	TNM Stage
HCC-1	Male	30	yes	T2	N0	M0	II
HCC-2	Male	50	yes	T1	N0	M0	I
HCC-3	Male	39	yes	T3a	N0	M0	IIIA
HCC-4	Male	40	yes	T2	N0	M0	II
HCC-5	Male	55	yes	T2	N0	M0	II
HCC-6	Male	52	no	T2	N0	M0	II
HCC-7	Male	41	yes	T2	N0	M0	II
HCC-8	Female	55	yes	T3b	N0	M0	IIIB
HCC-9	Male	54	yes	T1	N0	M0	I
HCC-10	Male	44	yes	T4	N0	M0	IIIC
HCC-11	Male	48	yes	T2	N0	M0	II
HCC-12	Male	59	yes	T4	N1	M0	IVA
HCC-13	Female	38	yes	T2	N0	M0	II
HCC-14	Male	47	yes	T3b	N0	M0	IIIB
HCC-15	Male	54	yes	T4	N1	M1	IVB

Supplementary Table 2: The sequence of primers

Name	Forward	Reverse
GAPDH	5'-TGTGTTGGCGTACAGGTCTTTG-3'	5'-GGGAAATCGTGCGTGACATTAAG-3'
PTTG3P	5'-ATGCTCCCACATCCTTACCTACT-3'	5'-AACGGGGTCCATTGGTCTTTA-3'
GBAP1	5'-GCATCATCACAAACCTCCGC-3'	5'-TCCTTCTTCTGACTGGCAACC-3'
CRNDE	5'-ATGGAAAAATCAAAGTGCTCGTG-3'	5'-CCTAACCTTCTTCTGCGTGACAAC-3'
LINC01419	5'-GCTTTGTTCTTTCGCTCTTCAT-3'	5'-TTCGTGGTCTCGCTGGCT-3'
CHL1-AS1	5'-GACTTCTCCCTACTTCGTTCACTGG-3'	5'-CCCGAGCATTTTTGTTTCTTCTG-3'
CYP2B7P	5'-CTGCCTCCTCCTACTGTTACCTT-3'	5'-CCCCCTTAGCGATGTTTATTTA-3'
H19	5'-ATCAAAGACACCATCGGAACAG-3'	5'-CAAGGTCAGAAACAAAGAGACAGAA-3'
NAPSB	5'-CACTACATCCCACCCCTCAAA-3'	5'-CAGTAAGCCCACCTTCACACG-3'
HAND2-AS1	5'-GCGATTTTGAAGTGCGGATA-3'	5'-ATAACAGGCGGTGGAGAGGAC-3'
A2M-AS1	5'-TGAAGCCTACTCAGACCGCA-3'	5'-CTACCGTTCCCAATAGAATACCAA-3'
LOC200772	5'-GAACCATACATTCAACGCAAACC-3'	5'-ACTCATCCAAAGGAGAAACAACC-3'
LINC01093	5'-AAGTCAGTGGTCCCTAAACGG-3'	5'-GAGCGGCAAAAGATAAACTCATAAG-3'
LINC01146	5'-TGATTCTGCTTACTGCTTCCCC-3'	5'-ATCCCTCCCCACTCTCTTCTAC-3'
LOC101928505	5'-TTGTGTTCATTCTCATCCCGC-3'	5'-CACTATCTCTCCTGCCACCTTGT-3'
